# *Amphiregulin* is overexpressed in human cardiac tissue in hypothermia deaths; associations between the transcript and stress hormone levels in cardiac deaths

**DOI:** 10.1080/07853890.2024.2420862

**Published:** 2024-11-07

**Authors:** Katja Porvari, Kie Horioka, Helena Kaija, Lasse Pakanen

**Affiliations:** a Research Unit of Biomedicine and Internal Medicine, Department of Forensic Medicine, Medical Research Center Oulu, University of Oulu, Oulu, Finland; b Forensic Medicine Unit, Finnish Institute for Health and Welfare, Oulu, Finland

**Keywords:** Amphiregulin, hypothermia and cardiovascular deaths, postmortem hypothermia marker, cardiac stress, ischaemia, catecholamines, cortisol

## Abstract

**Background:**

Amphiregulin (AREG) is a growth factor linked to cardioprotection and heart pathology during myocardial stress. Our aim was to investigate cardiac *AREG* expression, its potential as a postmortem hypothermia marker and its possible stress hormone dependency in different types of deaths.

**Materials and methods:**

Heart RNA was isolated from hypothermic, cardiac and non-cardiac deaths. Relative *AREG* mRNA levels and urine stress hormone concentrations were measured by qPCR and enzyme-linked immunosorbent assays from eight different death cause groups. Receiver operating characteristic curve was used to evaluate a cut-off point for *AREG* expression as a hypothermia marker. Regulatory elements were predicted by PROMO.

**Results:**

The *AREG* mRNA levels were significantly higher in hypothermic deaths than in most cardiac and non-cardiac deaths. *AREG* expression indicated hypothermic deaths with nearly 70% sensitivity and specificity. However, high expression levels were also detected in non-ischaemic deaths. The highest concentrations of adrenaline and cortisol were detected in hypothermic deaths, while the highest noradrenaline concentrations associated with atherosclerotic heart disease (AHD) deaths with acute myocardial infarction and trauma deaths. There were no significant correlations between stress hormones and *AREG* mRNA in hypothermic and non-cardiac deaths, whereas moderate-to-high associations were detected in cardiac deaths. Putative response elements for cortisol and catecholamines were found in *AREG*.

**Conclusions:**

Severe hypothermia activates cardiac *AREG* expression practicable as a postmortem hypothermia marker. Cortisol and catecholamines may act as transcriptional modifiers of this gene, especially in long-term ischaemic heart disease. However, the exact role of these hormones in upregulation of *AREG* during hypothermia remains unclear.

## KEY MESSAGES

- Amphiregulin appears to play a role in both protective and harmful cardiac events during acute cold stress and chronic ischaemic stress, respectively.
- Adrenaline, noradrenaline and cortisol can act as transcriptional regulators of *amphiregulin,* particularly during oxidative stress associated with AHD.
- High level of cardiac *amphiregulin* mRNA seems to be a sign of antemortem hypothermia.

## Introduction

Amphiregulin (AREG) [1] is a cytokine that belongs to the epidermal growth factor family. It is a transmembrane protein that requires proteolytic processing [2] before it acts as an autocrine, paracrine or juxtacrine factor through the epidermal growth factor receptor (EGFR) [3]. Thus, AREG activates intracellular signalling cascades that control cell proliferation, survival and motility [4]. The development and maturation of mammary glands [5], bone tissue [6] and oocytes [7] are examples of physiological roles of AREG. Furthermore, it is an essential cardioprotective mediator during myocardial ischaemia and adaptation to cardiac stress [8,9]. On the other hand, the excessive effect of AREG is linked to many pathological conditions, such as inflammation and/or neoplasia as well as cardiac fibrosis after myocardial infarction [10].

*AREG* gene is activated by hypothermia, as observed in our previous study, clarifying the effects of cold stress in healthy rat prostate tissue [11]. In addition, this gene was markedly upregulated during cold exposure involving cardiopulmonary bypass (CPB) and cardioplegic arrest in patients with diabetes [12]. Thus, increased expression of *AREG* could be a useful indicator of antemortem hypothermia or cold exposure in investigations of the cause of death (COD). The effect of cold on death is sometimes challenging to confirm owing to the lack of specific diagnostic markers or detectable signs in the body. Traditionally, the presence of small gastric mucosal lesions [13], high urine catecholamine concentrations or a high adrenaline (A) to noradrenaline (NA) ratio [14] have been considered to indicate hypothermia deaths. In addition to catecholamines, cortisol is a well-known stress hormone that shows increased levels in autopsy samples of hypothermia victims [15,16].

The mechanisms underlying cold-induced *AREG* activation are unknown. However, cyclic-AMP (cAMP) induces *AREG* transcription in many cell types [17], and both catecholamines and cortisol increase cAMP levels [18,19]. The promoter area of *AREG* contains a cAMP response element (CRE)-binding site [4], a mediator of cAMP effects on gene expression, and is a prospective cold stress-linked regulatory element for catecholamines and cortisol. According to our hypothesis, *AREG* is upregulated in the heart during severe hypothermia, and catecholamines, as well as cortisol, could be key regulators of this gene during cold stress.

Here, we measured human cardiac *AREG* transcript levels in hypothermic, cardiovascular disease (CVD) and non-cardiac deaths. The value of relative *AREG* expression as a marker of antemortem hypothermia was evaluated. The association between *AREG* expression and catecholamine and cortisol levels was investigated in relation to the COD. Putative glucocorticoid response elements (GREs) were screened in *AREG* regulatory area. The potential stress-connected regulatory mechanisms underlying *AREG* expression are discussed.

## Materials and methods

### Autopsy cases and sampling

Adhering to the Declaration of Helsinki [20], samples were collected as part of the routine medicolegal autopsies in the Department of Forensic Medicine, University of Oulu, Finland, and the Finnish Institute for Health and Welfare, Oulu, Finland. Myocardial samples were taken from the anterior wall of the left ventricle and urine samples were taken directly from the bladder. The samples were stored at −80 °C before analysis.

The total number of hypothermia cases in this study was 76; 54 of them had hypothermia as the primary COD (Group 1) and 22 as a contributing COD (Group 2) (Table 1). Of the 113 ischaemic CVD cases, 37 had atherosclerotic heart disease (AHD) as the primary COD (Group 3), 44 AHD with acute myocardial infarction (AMI) as immediate COD (Group 4) and 32 AHD with arterial hypertension (HTA) (Group 5). In addition, there were 7 non-ischaemic CVD deaths (Group 6), 20 trauma deaths (Group 7) and 31 cases classified as other deaths (Group 8). Group 6 included cadavers with hypertensive cardiomyopathy, cardiomegaly and cardiac amyloidosis. Group 8 consisted mainly of cadavers with ethanol intoxication, asphyxiation and drowning. Groups 7 and 8 represented non-cardiac deaths.

**Table 1.** Descriptives of the groups and number of cases in *AREG*, catecholamine and cortisol analyses.

Group no.	Cause of death	Age (year; mean + SE)	Sex (*n*; males/females)	PMI (days; median, range)	*AREG* mRNA	Catecholamines	Cortisol
					*n*	*n*	*n*
1	Hypothermia main cause	62.7 ± 2.3	36/18	7.5 (1–97)	54	54	20
2	Hypothermia contributory cause	64.4 ± 3.5	19/3	8.0 (2–16)	22	22	5
3	Ischaemic CVD/AHD	66.5 ± 2.3	32/5	4.0 (1–13)	37	15	15
4	Ischaemic CVD/AHD + AMI	65.0 ± 1.7	39/5	3.0 (1–10)	44	4	–
5	Ischaemic CVD/AHD + HTA	68.1 ± 1.5	25/7	4.0 (1–15)	32	11	9
6	Non-ischaemic CVD	68.8 ± 3.9	6/1	9.0 (4–14)	7	7	3
7	Trauma	34.2 ± 4.1	17/3	5.0 (2–35)	20	13	5
8	Other	53.5 ± 3.6	23/8	4.0 (0–55)	31	18	7
	All groups	61.3 ± 1.1	197/50	5.0 (0–97)	247	144	64

Abbreviations: CVD, cardiovascular disease; AHD, atherosclerotic heart disease; AMI, acute myocardial infarction; HTA, arterial hypertension; SE, standard error.

### AREG expression

Total RNA was extracted from myocardium samples (*n* = 247) using the miRNeasy mini kit (Qiagen, Hilden, Germany) and an automated QIAcube sample preparation instrument (Qiagen, Hilden, Germany) according to the manufacturer’s protocol. A High-Capacity cDNA Reverse Transcription Kit (Applied Biosystems, Foster City, CA, USA) was used to reverse-transcribe the RNAs with random primers according to the manufacturer’s protocol.

The cDNAs were amplified with a Rotor-Gene Q (Qiagen) using gene-specific primers (Sigma, Haverhill, UK) and Maxima SYBR Green qPCR Master Mix (Thermo Scientific) according to the manufacturer’s protocols. The primer sequences for human *AREG* were 5′-GAGCACCTGGAAGCAGTAACAT-3′ and 5′-GGACTTTTCCCCACACCGTTCA-3′ (GenBank accession number NM_001657.4, amplicon size 69 bp). The primer sequences for human *GAPDH* were 5′-TGGAAGGACTCATGACCACA-3′ and 5′-CCATCACGCCACAGTTT-3′ (GenBank accession number BC029618, amplicon size 85 bp).

The amplification was carried out as follows: 1 cycle of denaturation (95 °C for 10 min) followed by 40 cycles of three-stage PCR (95 °C for 15 s, 60 °C for 30 s and 72 °C for 30 s). Fluorescence signals were continuously measured during repetitive cycles. *AREG* expression was normalized to the reference gene *GAPDH* using the 2^−ΔΔCt^ method [21]. Commercial human myocardial total RNA (Clontech, Mountain View, CA, USA) pooled from normal hearts of three Caucasian males (ages between 30 and 39 years; trauma as the COD) was used as a reference sample. The relative expression of AREG was obtained by comparing the normalized expression to that of the commercial reference sample, which was given a value of 1.

### *AREG* in situ *hybridization and IHC*

Sections of human FFPE heart tissue specimens were stained with manual RNAscope 2.5 HD Red Assay (ACD, Bio-Techne Ltd, UK) according to the manufacturer’s instructions using *AREG* probe. Dewaxed and rehydrated tissue sections were pretreated with RNAscope Target Retrieval (98 °C, 15 min) and Protease Plus (RT, 30 min).

Immunohistochemical staining was performed by Leica Bond RX automated using a Refine Detection Kit with 1:100 dilution of monoclonal AREG antibody G-4 (SC-74501). Heat-induced epitope retrieval at pH 6 was performed before staining dewaxed and rehydrated heart tissue sections.

### Catecholamine and cortisol measurements

Catecholamine concentrations were analysed in urine samples (*n* = 144) using an enzyme-linked immuno­sorbent assay (ELISA; Cat Combi ELISA kit, DRG International, Inc., Springfield, NJ, USA). According to the manufacturer, the analytical sensitivities of A and NA were 0.011 and 0.044 ng/ml, respectively. Coefficients of variance for intra-assay precision were 15.0% for A and 16.1% for NA at 2.5 and 24.4 ng/ml levels, respectively.

Cortisol concentrations were measured in urine samples (*n* = 64) using a Cortisol ELISA kit (Enzo Life Sciences, Inc. NY, USA). According to the manufacturer, the detection limit of the assay was 56.72 pg/ml. Coefficients of variance for intra- and inter-assay precisions for high cortisol concentrations (3155/3052 pg/ml) were 7.3% and 8.6%, respectively.

### Statistics

Statistical analyses were performed using IBM SPSS Statistics version 24 (Armonk, NY, USA). The Shapiro–Wilk and Kolmogorov–Smirnov tests were used to test the normality of the data. Only part of the data followed a normal distribution. In addition, the size of the groups was small. For these reasons, pair-wise comparisons between the groups were made using the non-parametric Mann–Whitney test (statistical significance level set at *p* < 0.05). The non-parametric Spearman’s rho correlation test was used to analyse the associations between the selected analytes and correlations were visualized by scatterplots using the GraphPad Prism.

### Prediction of transcription factor binding sites on AREG promoter

Glucocorticoid receptor (GR) binding sites (factor/matrix/width: GR/T05076/7; GR-alpha/T00337/5 and GR-beta/T01920/5) and sites for cAMP response element-binding protein (CREB/T00163/9) were predicted by PROMO [22,23] using the TRANSFAC database version 8.3. Predictions were carried out using 10% as the maximum dissimilarity margin and limited to proximal promoter of AREG, including 500 nucleotides upstream from the ATG codon.

## Results

### Autopsy cases

Characteristics of cadavers are described in Table 1: the mean age of the traumatic death group was lower than that of the other seven groups. The proportion of males in the groups varied from 66% to 88%, being lowest in the hypothermia main COD group (Group 1) and highest in the ischaemic CVD/AHD + AMI group (Group 4). Median values of postmortem intervals (PMIs) varied between 3 and 8.5 days in the groups. Among the hypothermia victims, 37% of Group 1 and 64% of Group 2 cases had ischaemic CVD/AHD as a contributory COD.

### AREG transcript levels in myocardium

**Figure 1.**
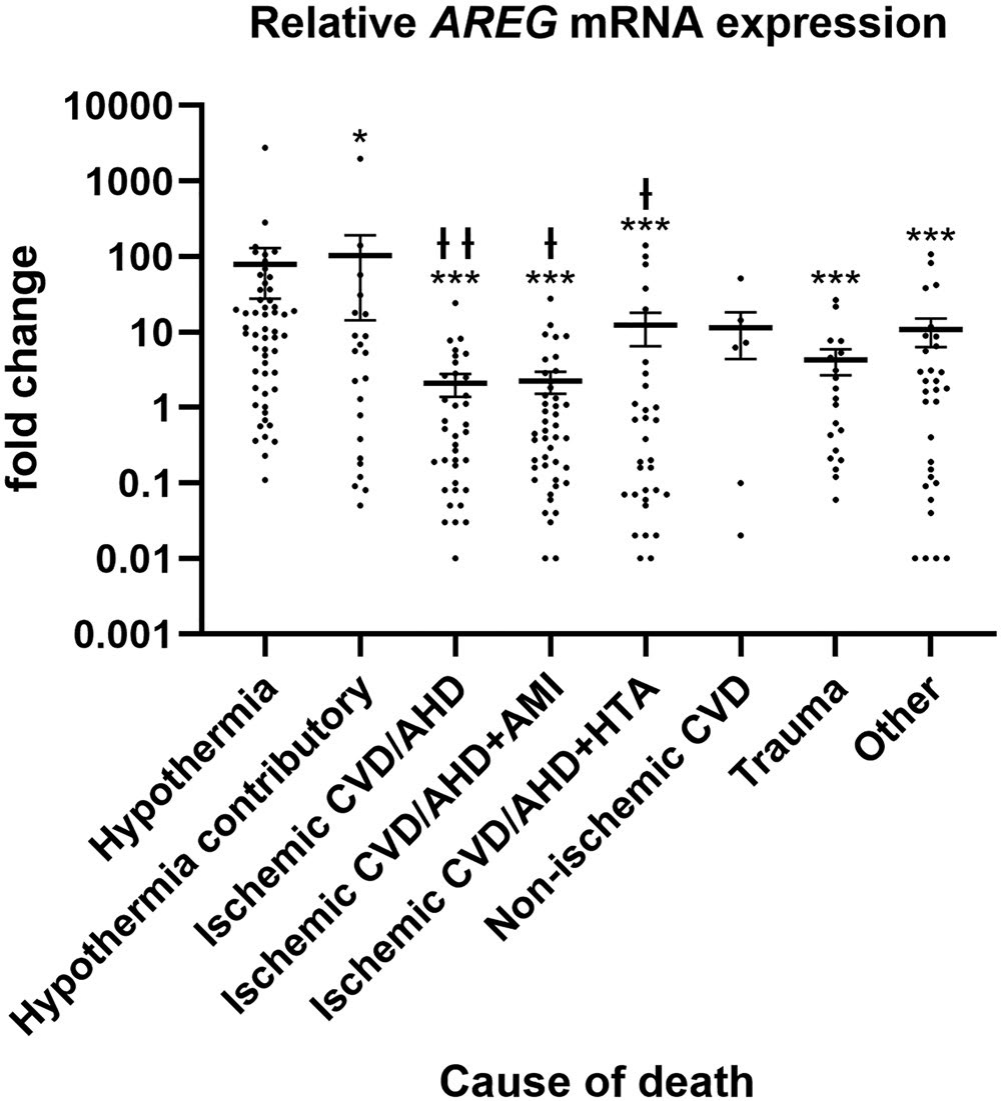
*Amphiregulin* expression in human heart tissue in different types of deaths. Mean relative *AREG* mRNA level is shown with standard error and including individual data points on logarithmic scale. COD, cause of death; CVD, cardiovascular disease; AHD, atherosclerotic heart disease; AMI, acute myocardial infarction; HTA, arterial hypertension. *p < 0.05, ***p < 0.001 vs. hypothermia main COD; ^Ɨ^*p* < 0.05, ^Ɨ Ɨ^*p* < 0.01 vs. hypothermia contributory COD.

*AREG* expression was measured by qPCR relative to *GAPDH* expression. The mean (±SE) relative *AREG* mRNA expression in heart tissue was 79 (±51) for hypothermia main COD, 104 (±89) for hypothermia contributory COD, approximately 2 (±1) for both ischaemic CVD/AHD and CVD/AHD + AMI groups, 12 (±6) for ischaemic CVD/AHD + HTA, 11 (±7) for non-ischaemic CVD, 4 (±2) for trauma and 11 (±4) for other deaths (Figure 1). The *AREG* transcript level in the hypothermia main COD group was significantly higher than that in all non-hypothermia death groups, except in Group 6, which represents non-ischaemic CVD deaths. The *AREG* mRNA level in the hypothermia contributory COD group (Group 2) was even higher than that in the hypothermia main COD group, which was also significantly higher than that in the three ischaemic CVD groups (Groups 3–5). The differences between the CVD groups (Groups 3–6) were not significant.

Possible dependency of *AREG* expression on cadaver’s sex, age and PMI was determined. When analysing all cases, the mean (±SE) relative *AREG* mRNA level was 33 (±17) for male and 20 (±7) for female cadavers, the difference being statistically significant (*p* < 0.05). *AREG* expression did not correlate with age at death or PMI. Thus, the expression differences between the COD groups do not originate from age or PMI differences, but sex should be considered as a confounding factor.

The potential of the relative cardiac *AREG* mRNA expression level to distinguish hypothermic deaths from other types of deaths was analysed using an Receiver operating characteristic curve. The area under the curve was 0.767 (*p* < 0.001), indicating that the expression level of 2.2 was the optimal cut-off point. The sensitivity to detect hypothermia with expression level greater than or equal to the cut-off point was 69.7%, while the specificity of the assay was 69.6%. Using this cut-off point, 57.1% of the non-ischaemic deaths represented high *AREG* expression levels, while the corresponding number was maximally around 45% in other non-hypothermic COD groups (Table 2).

**Table 2.** Distribution of samples according to relative *AREG* mRNA cut-off value in hypothermic, cardiac and non-cardiac deaths.

Groups	Cases/group	Relative *AREG* mRNA expression			
		Low (<2.2)		High (≥2.2)	
	*n*	*n*	%	*n*	%
1 Hypothermia main COD	54	14	25.9	40	74.1
2 Hypothermia contributory COD	22	9	40.9	13	59.1
3 Ischaemic CVD/AHD	37	27	73.0	10	27.0
4 Ischaemic CVD/AHD + AMI	44	35	79.5	9	20.5
5 Ischaemic CVD/AHD + HTA	32	25	78.1	7	21.9
6 Non-ischaemic CVD	7	3	42.9	4	57.1
7 Trauma	20	12	60.0	8	40.0
8 Other	31	17	54.8	14	45.2
*All hypothermia deaths*	76	*23*	*30.3*	*53*	*69.7* a
*All non-hypothermia deaths*	171	*119*	*69.6* b	*52*	*30.4*

Abbreviations: COD, cause of death; CVD, cardiovascular disease; AHD, atherosclerotic heart disease; AMI, acute myocardial infarction; HTA, arterial hypertension.

^a^Sensitivity.

^b^Specificity.

### Localization of AREG transcripts and protein in heart tissue

**Figure 2.**
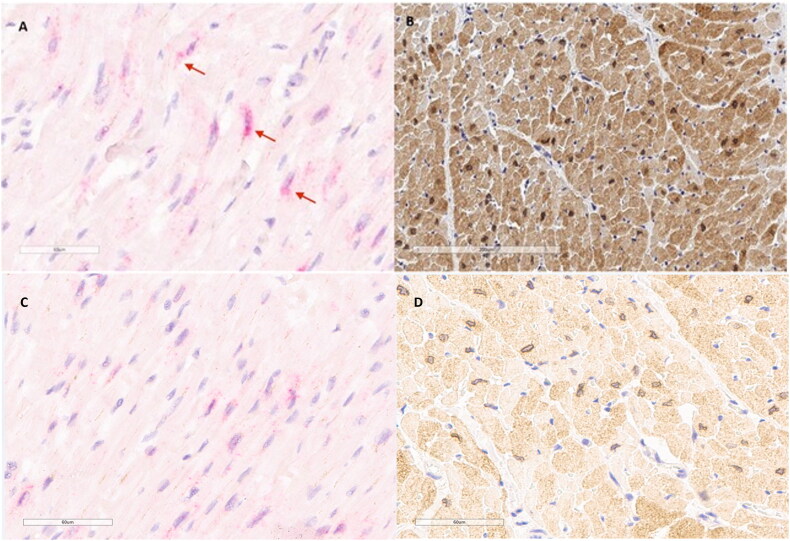
Localization of amphiregulin in the human heart from hypothermia victim. (A,C) *AREG* transcripts (red dots) are present in cytoplasm and in some nuclei of cardiomyocytes. (B,D) Signal for AREG protein is detected in cardiomyocytes with cytoplasmic staining and membranous staining of nuclei.

According to *in situ* hybridization assay *AREG* transcripts were mainly detected in the cytoplasm of cardiomyocytes in the heart of hypothermia victim (Figure 2A,C). Positive signals were also observed in some of the nuclei. According to immunohistochemistry staining AREG protein was present in the cytoplasm of cardiomyocytes, showing also membranous staining around the nuclei (Figure 2B,D).

### Urine catecholamine levels and correlation with cardiac AREG expression

**Figure 3.**
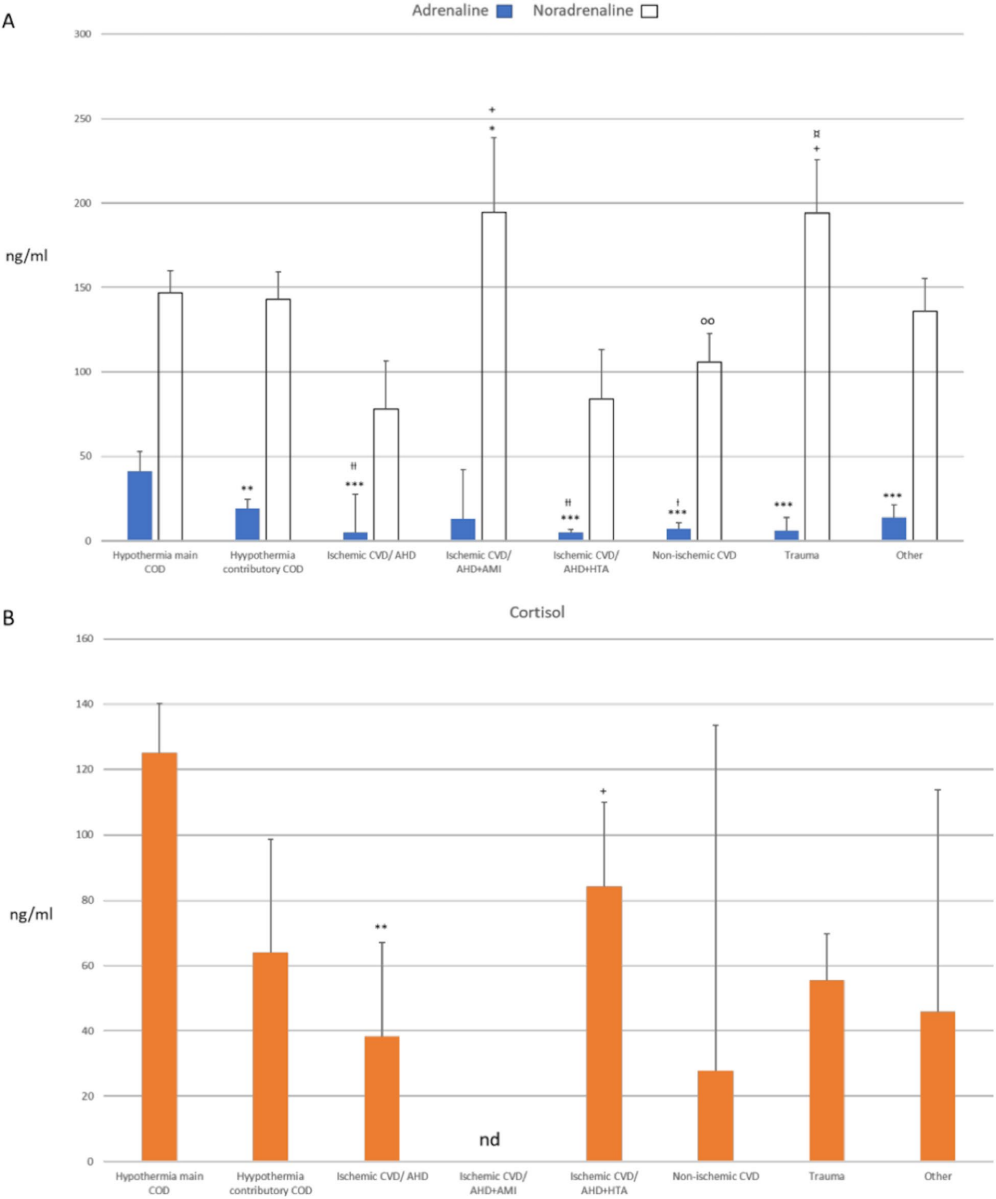
Urine concentrations of the stress hormones in different death cause groups. Median level (ng/ml) with standard error for A and NA (A) and cortisol (B). COD, cause of death; CVD, cardiovascular disease; AHD, atherosclerotic heart disease; AMI, acute myocardial infarction; HTA, arterial hypertension. *p < 0.05, **p < 0.01, ***p < 0.001 vs. hypothermia main COD; ^Ɨ^*p* < 0.05, ^Ɨ Ɨ^*p* < 0.01 vs. hypothermia contributory COD; ^+^*p* < 0.05 vs. ischaemic CVD/AHD; ^oo^*p* < 0.01 vs. CVD/AHD + AMI; ^¤^*p* < 0.05 vs. CVD/AHD + HTA.

Catecholamine levels were measured using the specific ELISA method. The median A and NA concentrations in different death cause groups are shown in Figure 3A, and the highest A levels were detected in hypothermia and hypothermia-contributory cases. In contrast, the highest NA levels were found in ischaemic CVD/AHD + AMI (Group 4) and trauma (Group 7) deaths.

**Figure 4.**
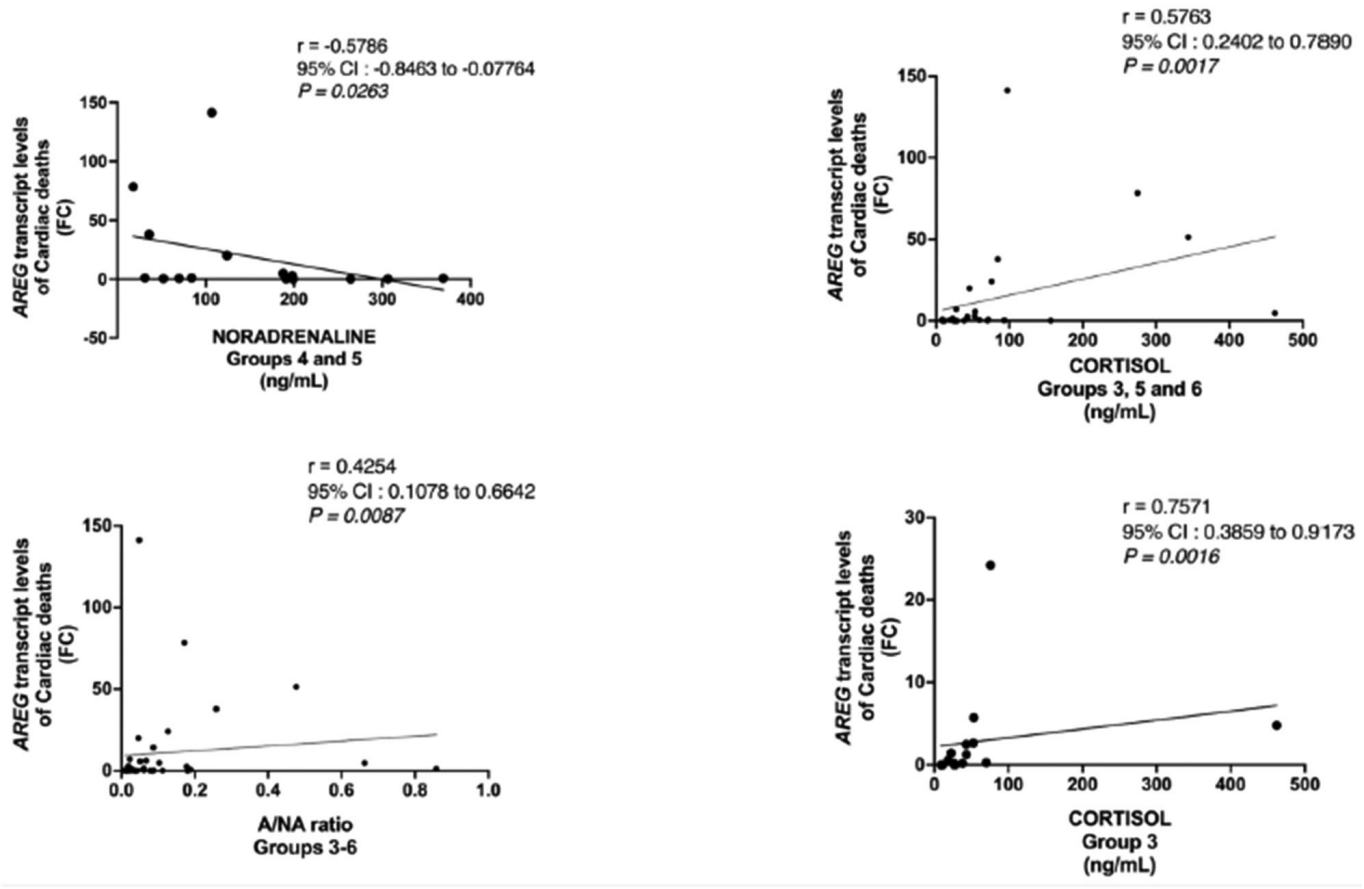
Visualization of correlations between *amphiregulin* expression and stress hormones. The non-parametric Spearman’s rho correlation tests were carried out and presented as scatterplots using the GraphPad Prism. FC, fold change; CI, confidence interval; A/NA, adrenaline to noradrenaline ratio.

To analyse possible catecholamine dependency of *AREG* expression, correlation studies were carried out. Significant correlations between the relative *AREG* mRNA expression in heart tissue and urine A concentration (*r* = 0.297, *p* < 0.01*)* or A/NA ratio (*r* = 0.333, *p* < 0.01) were observed when all available cases were combined (*n* = 143). *AREG* mRNA and A/NA levels were also correlated in the combined CVD cases (Groups 3–6, *n* = 37; *r* = 0.425, *p* < 0.01; Table 3). The only significant correlation between urine NA concentration and the *AREG* mRNA (*r* = −0.579, *p* < 0.05) was found in the combined ischaemic CVD/AHD groups 4 and 5 (*n* = 15), which included cases with AMI or HTA. Correlations are visualized in scatterplots (Figure 4). Correlations between urine catecholamines and cardiac *AREG* expression were not detected in hypothermic or non-cardiac deaths.

**Table 3.** Correlations between the relative cardiac *AREG* transcript levels and urine catecholamines or cortisol concentration associated to COD.

	Relative *AREG* transcript levels in heart		
	Hypothermic deaths (Groups 1 and 2)	Cardiac deaths(Groups 3–6)	Non-cardiac deaths (Groups 7 and 8)
A	−a	–	–
NA	–	−0.579*^b^	–
A:NA ratio	–	0.425**^c^	–
Cortisol	–	0.576**^d^	–
Cortisol	–	0.757**^e^	–

^a^–, not significant.

^b^*, p < 0.05 in combined Groups 4 and 5 (*n* = 15).

^c^**, p < 0.01 in combined Groups 3–6 (*n* = 37).

^d^In combined Groups 3, 5 and 6 (*n* = 27).

^e^In Group 3 (*n* = 15).

### Urine cortisol levels and correlation with cardiac AREG expression

Cortisol levels were measured using the specific ELISA method. The median cortisol concentrations in the different death groups are shown in Figure 3B. The highest concentration was detected in hypothermia deaths (Group 1), which was significantly higher than that in ischaemic CVD/AHD deaths (Group 3). Furthermore, the cortisol concentration in CVD/AHD + HTA deaths (Group 5) was significantly higher than that in CVD/AHD deaths.

To analyse possible cortisol dependency of *AREG* expression, correlation studies were carried out. A significant correlation between relative *AREG* mRNA expression in heart tissue and urine cortisol concentration (*r* = 0.395, *p* < 0.01) was observed when all available cases were combined (*n* = 64). Similarly, a significant correlation was observed in the combined CVD cases (Groups 3, 5 and 6, *n* = 27; *r* = 0.576, *p* < 0.01; Table 3). The highest correlation coefficient (*r* = 0.757, *p* < 0.01) was detected in the ischaemic CVD/AHD group (Group 3, *n* = 15). Scatterplots for correlations are presented in Figure 4. No significant correlation was found between the relative *AREG* mRNA expression and urine cortisol concentration in the hypothermia groups or non-cardiac deaths.

### Mutual relationships between urine cortisol and catecholamines in relation to COD

**Figure 5.**
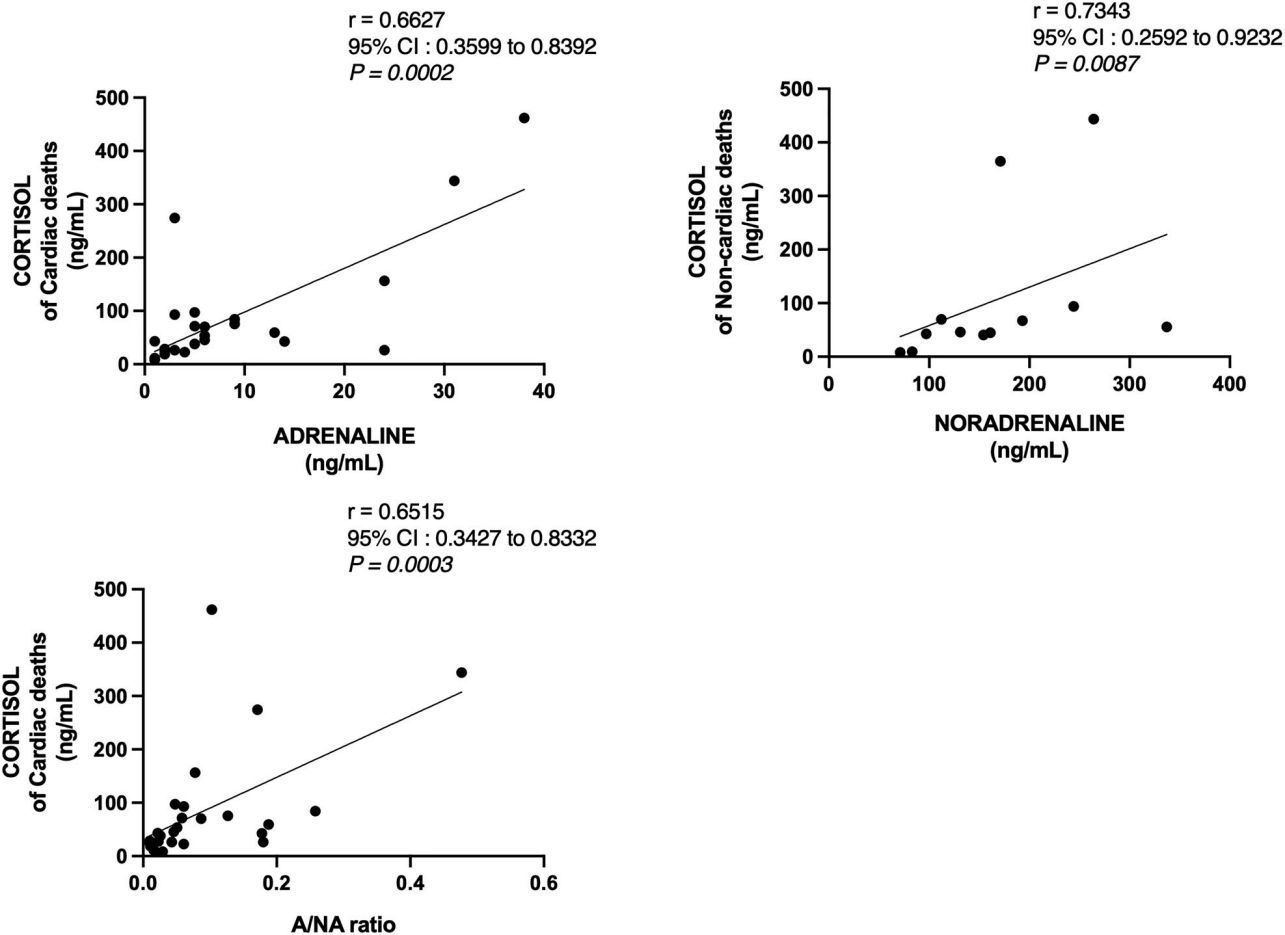
Visualization of correlations between catecholamines and cortisol. The non-parametric spearman’s rho correlation tests were carried out and presented as scatterplots using the GraphPad Prism. CI, confidence interval; A/NA, adrenaline to noradrenaline ratio.

Correlations between the stress hormones in different COD groups were evaluated. There was a lack of significant correlation between urine cortisol and catecholamine levels in hypothermic deaths (Table 4). In contrast, both A (*r* = 0.663, *p* < 0.001) and the A/NA ratio (*r* = 0.652, *p* < 0.001) correlated with cortisol concentrations in cardiac deaths. A strong correlation between cortisol and NA concentrations was detected exclusively in non-cardiac deaths (Groups 7 and 8; *r* = 0.734, *p* < 0.01). Correlations are visualized in scatterplots (Figure 5).

**Table 4.** Correlations between urine cortisol and catecholamine concentrations related to COD.

	Cortisol		
	Hypothermic deaths (Groups 1 and 2; *n* = 25)	Cardiac deaths(Groups 3, 5, 6; *n* = 27)	Non-cardiac deaths (Groups 7 and 8; *n* = 12)
A	−^a^	0.663***^b^	–
NA	–	–	0.734**^c^
A:NA ratio	–	0.652***	–

^a^–, not significant.

^b^***, *p* < 0.001.

^c^**, *p* < 0.01.

### Putative glucocorticoid receptor binding sites on AREG promoter

**Figure 6.**
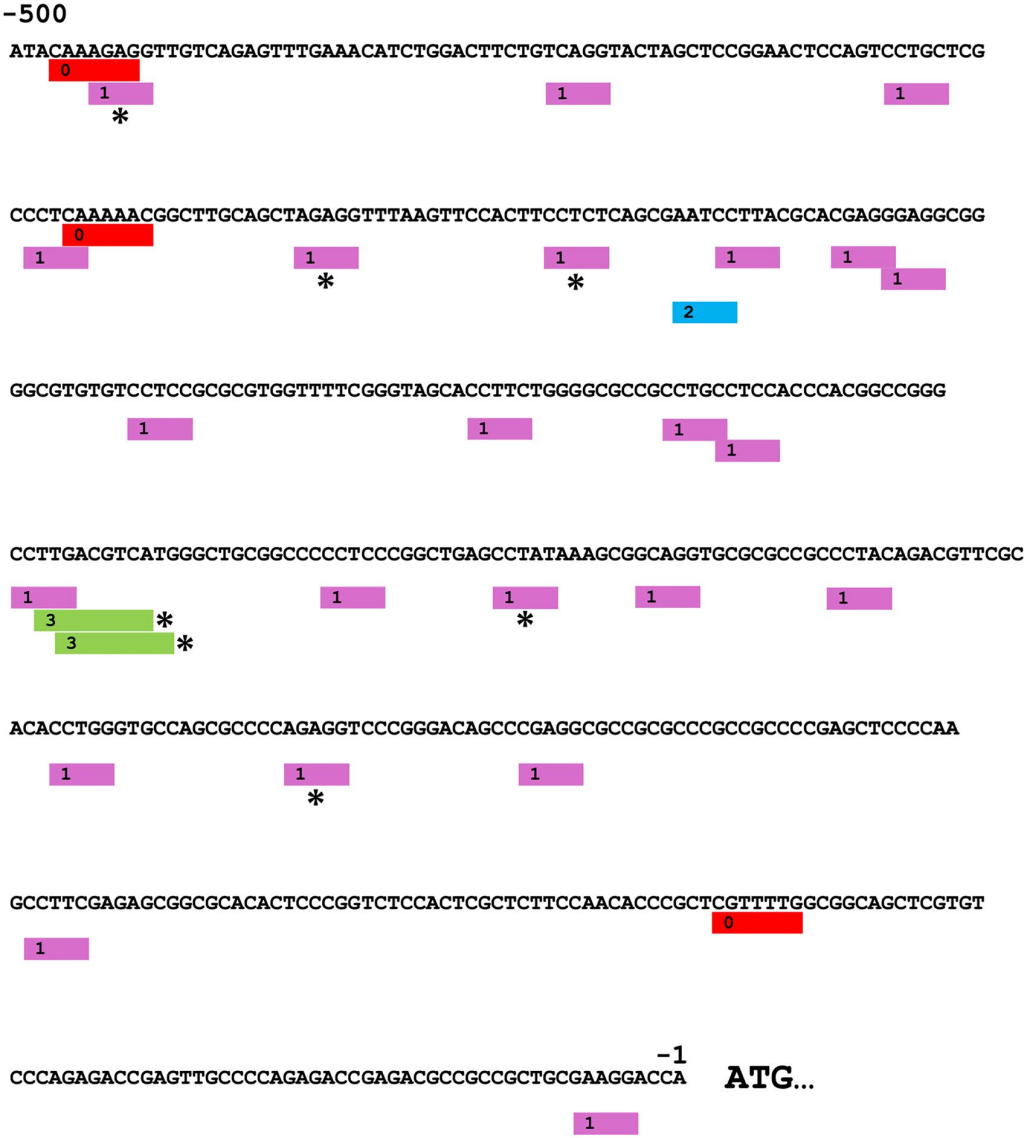
Potential regulatory elements for catecholamines and cortisol in *AREG* proximal promoter. Selected transcription factor binding sites were predicted by PROMO using TRANSFAC database. Putative GR binding sites and sites for CREB are shown along the *AREG* sequence 500 bp upstream from ATG-codon. Blocks marked with 0, 1 and 2 represent putative GR, GR-alpha and GR-beta binding sites, respectively. Blocks marked with 3 represent CREB binding sites. Sites marked with * have dissimilarity margins ≤0.21% and others ≤10%.

Putative transcription factor binding sites were predicted by PROMO program. Several putative binding sites for GRs were found with 10% dissimilarity margin in *AREG* proximal promoter (Figure 6). Most of these elements represent GR-alpha type sites, and five of them had dissimilarity margins as low as 0.00 − 0.21%. Only one GR-beta and three GR sites were found in the promoter sequence. All except one overlapped with the GR-alpha sites. Also, two overlapping CRE elements (CREB) with functional evidence of cAMP response [9] were located on the promoter, showing 0.11% and 0.07% dissimilarity margins.

## Discussion

Previously, we detected increased *AREG* transcript levels in the prostate tissues of cold-exposed and rewarmed healthy rats [11]. However, we could not observe cold-induced activation of *AREG* in the cardiac tissue of these rats (unpublished findings). The tissue-specific response is probably related to temperature differences in actively warmed hearts compared to peripheral prostatic tissues, which are more vulnerable to heat loss during cold exposure. In the current study, increased relative levels of human cardiac *AREG* mRNA were detected in hypothermia-related deaths. These results suggest that severe or fatal cold exposure is needed for *AREG* gene activation in the heart tissue. On the other hand, a 16-fold increase in *AREG* transcript levels was detected in atrial myocardium samples after mild hypothermic (33–35 °C) CPB in diabetic patients but not in non-diabetic controls. One explanation for this could be the increased oxidative stress in patients with diabetes [12].

Catecholamines are probably among the most suitable postmortem markers for hypothermia [24], with 69% sensitivity and 78% specificity when urine A to NA ratio is applied [14]. The sensitivity of the relative cardiac *AREG* mRNA level to detect hypothermia involved deaths was comparable to that of catecholamines, although with a bit lower specificity (70%). To date, most of the putative RNA-based markers expressed in heart tissue, such as *RNU6b* [25], *p21alt-a* [26], and natriuretic peptides *ANP* and *BNP* [27], show decreased levels in hypothermic deaths, whereas upregulated genes such as *AREG* could be more practical in investigations of death. In addition, some candidate genes identified in animal models could be beneficial in the diagnosis of fatal hypothermia in the future: *Ctgf*, *Junb, Nr4a1* and *Sdc4* are upregulated in cold-exposed iliopsoas muscle [28]. *Junb* is also upregulated in the mouse adrenal gland, together with several other potential hypothermia markers [29]. Furthermore, increased levels of several miRNAs are associated with severe hypothermia in muscle tissue [30]. Among these, *rno-miR-374-5p* seems to enhance the viability of iliopsoas muscle cells by regulating apoptosis-associated CASP3 and CASP6 proteins.

Activation of the sympathetic nervous system with increased levels of catecholamines and cortisol is the cornerstone of cold stress. A and NA act via the beta-adrenergic receptor (βAR) to increase cAMP levels and further activate the pKA signalling pathway in cardiac cells [18]. Generally, cortisol binds to the GR, which then interacts with GREs in specific genes to change their expression. In addition to this conventional type of genomic action, glucocorticoids have been shown to increase cellular cAMP production via G protein-coupled receptors, similar to catecholamines [19]. Along *AREG* proximal promoter, several putative GREs could have a regulatory role during hypothermia and other stress conditions. Although *AREG* expression is known to be increased by cAMP, we did not observe correlations between relative cardiac *AREG* mRNA levels and urine catecholamine or cortisol concentrations in hypothermic deaths. In contrast, moderate-to-strong associations were detected between these hormones and myocardial *AREG* transcript levels in cardiac deaths, most of which were associated with long-term ischaemic stress. However, our results do not necessarily exclude the possibility that *AREG* expression is upregulated by increased cAMP levels both in hypothermia and CVD-connected ischaemic stress, and induction of *AREG* seems to take place in severe or fatal stages of hypothermia when the correlation between the end-state transcript and hormone levels is undoubtedly challenging to detect. On the other hand, agonist-independent βAR dysfunction, like that described for hypoxia [31], might contribute to the lack of correlation between cold stress-connected hormones and *AREG* expression in hypothermic deaths.

In addition to catecholamines and cortisol, some other factors probably participate in the regulation of gene expression during cold stress. For example, hypothermia has been shown to induce a hypoxic response in torpor and hibernating animals [32]. Extreme hypothermia can also cause acute hypoxia in the human heart and may activate hypoxia-inducible transcription factors. Two HIF2 alpha-binding sites are present in the promoter of *AREG* [4], offering a feasible mechanism for the upregulation of this gene during hypothermia and simultaneous hypoxic stress. This might also describe a mechanism underlying hypothermia-induced protection of heart tissue, similar to the suggested HIF2A-AREG-ERBB1-pAkt dependent cardioprotection during myocardial ischaemia and reperfusion injury [9, 33].

Prolonged exposure to catecholamines and cortisol can lead to harmful cellular events. In line with this, our results show high NA and cortisol concentrations in the ischaemic CVD/AHD + AMI and CVD/AHD + HTA groups, respectively. AREG is associated with several fibrotic diseases including cardiac fibrosis after myocardial infarction [10] and cirrhotic cardiomyopathy [34]. Here, we showed a moderate positive correlation between the A/NA ratio and relative cardiac *AREG* mRNA levels in CVD cases (*r* = 0.425, *p* < 0.01). Interestingly, NA concentration was negatively correlated with the *AREG* transcript level exclusively in the combined ischaemic CVD/AHD groups, including cases with AMI or HTA (*r* = −0.579, *p* < 0.05). This might be due to the use of medication based on beta-adrenergic block to decrease blood pressure. Furthermore, a strong positive association between cortisol concentration and relative *AREG* expression was detected in cardiac deaths, especially in the CVD/AHD group (*r* = 0.757, *p* < 0.01). Thus, our results suggest that continuous exposure to catecholamines and cortisol could cause myocardial dysfunction among other ways via *AREG* expression. In addition, chronic catecholamine stimu­lation activates ADAM17 protease to release its membrane-bound fibrotic substrates, including AREG and tumor necrosis factor alpha [35].

Urine concentrations of cortisol and A correlated moderately in cardiac deaths, while NA and cortisol concentrations associated strongly in non-cardiac deaths. Interestingly, such correlations were not detected in hypothermic deaths. Nonetheless, cortisol secretion increases later than catecholamines in acute cold stress. In cardiac deaths, the mutual connection between urine A and cortisol concentrations could describe similar adaptive responses to prolonged sympathetic activation during chronic ischaemic stress. The association between NA and cortisol might illustrate the interplay of these hormones in acute stress, which is typical of non-cardiac deaths caused by trauma.

According to a recent study *AREG* downregulation opposes cardiac hypertrophy through reduction of oxidative stress and apoptosis [36]. It is possible that hypothermia induces lipoperoxidation [37,38], worsening oxidative damage in cardiomyocytes with dysfunction of mitochondria [39]. Thus, *AREG* upregulation in hypothermia-related deaths might correlate positively with cardiomyocyte death. Anyway, AREG is an extremely interesting target for novel clinical treatments as well as diagnostic purposes widely in medicine.

We showed here that the relative *AREG* mRNA expression in the human heart was significantly higher in hypothermia and hypothermia contributory deaths than in most cardiac or non-cardiac deaths, indicating its potential for use as a postmortem marker for hypothermia. High urine A and cortisol concentrations were detected in hypothermic deaths, while high NA levels were observed, especially in CVD/AHD + AMI and trauma deaths. There were significant associations between stress hormones and *AREG* expression in cardiac deaths but not in hypothermic or non-cardiac deaths.

## Conclusion

Our results suggest that *AREG* function is involved in both protective and harmful cardiac events during acute cold stress and chronic ischaemic stress, respectively. Catecholamines and cortisol can act as transcriptional regulators of *AREG,* particularly in oxidative stress associated with AHD. High level of cardiac *AREG* mRNA seems to indicate antemortem hypothermia.

## Strengths and limitations/studies in perspective

The strength of this study is the high number of human samples which improves the reliability of the expression results. Simultaneously, samples available for hormone assays were limited in some death groups and only associations between molecular connections were reported. To get direct evidence on the stress hormone dependency of *AREG* gene, animal and cell model experiments ought to be performed. Future studies should also include validation of the results and cases with widely approved markers for hypothermia, oxidative stress, hypertrophy, apoptosis and fibrosis.

## Data Availability

The data presented in this study are available upon reasonable request.

## References

[1] Shoyab M, McDonald VL, Bradley JG, et al. Amphiregulin: a bifunctional growth-modulating glycoprotein produced by the phorbol 12-myristate 13-acetate-treated human breast adenocarcinoma cell line MCF-7. Proc Natl Acad Sci U S A. 1988;85(17):6528–6532. doi: 10.1073/pnas.85.17.6528. 3413110 PMC282006

[2] Levano KS, Kenny PA. Clarification of the C-terminal proteolytic processing site of human Amphiregulin. FEBS Lett. 2012;586(19):3500–3502. doi: 10.1016/j.febslet.2012.07.078. 22967896

[3] Johnson GR, Kannan B, Shoyab M, et al. Amphiregulin induces tyrosine phosphorylation of the epidermal growth factor receptor and p185erbB2. Evidence that amphiregulin acts exclusively through the epidermal growth factor receptor at the surface of human epithelial cells. J Biol Chem. 1993;268(4):2924–2931. ISSN 00219258. doi: 10.1016/S0021-9258(18)53862-X. 7679104

[4] Berasain C, Avila MA. Amphiregulin. Semin Cell Dev Biol. 2014;28:31–41. PMID: 24463227. doi: 10.1016/j.semcdb.2014.01.005. 24463227

[5] Luetteke NC, Qiu TH, Fenton SE, et al. Targeted inactivation of the EGF and amphiregulin genes reveals distinct roles for EGF receptor ligands in mouse mammary gland development. Development. 1999;126(12):2739–2750. PMID: 10331984. doi: 10.1242/dev.126.12.2739. 10331984

[6] Qin L, Tamasi J, Raggatt L, et al. Amphiregulin is a novel growth factor involved in normal bone development and in the cellular response to parathyroid hormone stimulation. J Biol Chem. 2005;280(5):3974–3981. PMID: 15509566. doi: 10.1074/jbc.M409807200. 15509566

[7] Zamah AM, Hsieh M, Chen J, et al. Human oocyte maturation is dependent on LH-stimulated accumulation of the epidermal growth factor-like growth factor, amphiregulin. Hum Reprod. 2010;25(10):2569–2578. doi: 10.1093/humrep/deq212. 20719813 PMC2939758

[8] Fujiu K, Shibata M, Nakayama Y, et al. A heart-brain-kidney network controls adaptation to cardiac stress through tissue macrophage activation. Nat Med. 2017;23(5):611–622. PMID: 28394333. doi: 10.1038/nm.4326. 28394333

[9] Lee JW, Koeppen M, Seo SW, et al. Transcription-independent induction of ERBB1 through hypoxia-inducible factor 2A provides cardioprotection during ischemia and reperfusion. Anesthesiology. 2020;132(4):763–780. doi: 10.1097/ALN.0000000000003037. 31794514 PMC7072004

[10] Liu L, Song S, Zhang YP, et al. Amphiregulin promotes cardiac fibrosis post myocardial infarction by inducing the endothelial-mesenchymal transition via the EGFR pathway in endothelial cells. Exp Cell Res. 2020;390(2):111950. Erratum in: Exp Cell Res. 2023;429(2):113673. PMID: 32188578. doi: 10.1016/j.yexcr.2020.111950. 32188578

[11] Kaija H, Pakanen L, Kortelainen M-L, et al. Hypothermia and rewarming induce gene expression and multiplication of cells in healthy rat prostate tissue. PLoS One. 2015;10(5):e0127854. doi: 10.1371/journal.pone.0127854. 25996932 PMC4440734

[12] Voisine P, Ruel M, Khan TA, et al. Differences in gene expression profiles of diabetic and nondiabetic patients undergoing cardiopulmonary bypass and cardioplegic arrest. Circulation. 2004;110(11 Suppl 1):II280–II286. PMID: 15364876. doi: 10.1161/01.CIR.0000138974.18839.02. 15364876

[13] Takada M, Kusano I, Yamamoto H, et al. Wischnevsky’s gastric lesions in accidental hypothermia. Am J Forensic Med Pathol. 1991;12(4):300–305. doi: 10.1097/00000433-199112000-00006. 1807139

[14] Pakanen L, Kortelainen M-L, Särkioja T, et al. Increased adrenaline to noradrenaline ratio is a superior indicator of antemortem hypothermia compared with separate catecholamine concentrations. J Forensic Sci. 2011;56(5):1213–1218. doi: 10.1111/j.1556-4029.2011.01805.x. 21595691

[15] Nishio T, Toukairin Y, Hoshi T, et al. Relationships between cause of death and concentrations of seven steroids obtained from the serum and cerebrospinal fluid of cadavers. J Forensic Leg Med. 2023;96:102516. PMID: 37011448. doi: 10.1016/j.jflm.2023.102516. 37011448

[16] Shida A, Ikeda T, Tani N, et al. Cortisol levels after cold exposure are independent of adrenocorticotropic hormone stimulation. PLoS One. 2020;15(2):e0218910. doi: 10.1371/journal.pone.0218910. 32069307 PMC7028257

[17] Johansson CC, Yndestad A, Enserink JM, et al. The epidermal growth factor-like growth factor amphiregulin is strongly induced by the adenosine 3′,5′-monophosphate pathway in various cell types. Endocrinology. 2004;145(11):5177–5184. PMID: 15284208. doi: 10.1210/en.2004-0232. 15284208

[18] Motiejunaite J, Amar L, Vidal-Petiot E. Adrenergic receptors and cardiovascular effects of catecholamines. Ann Endocrinol. 2021;82(3–4):193–197. PMID: 32473788. doi: 10.1016/j.ando.2020.03.012. 32473788

[19] Nuñez FJ, Johnstone TB, Corpuz ML, et al. Glucocorticoids rapidly activate cAMP production via G_αs_ to initiate non-genomic signaling that contributes to one-third of their canonical genomic effects. FASEB J. 2020;34(2):2882–2895. doi: 10.1096/fj.201902521R. 31908022 PMC7027561

[20] World Medical Association. World Medical Association Declaration of Helsinki: ethical principles for medical research involving human subjects. JAMA. 2013;310(20):2191–2194. doi: 10.1001/jama.2013.281053. 24141714

[21] Livak KJ, Schmittgen TD. Analysis of relative gene expression data using real-time quantitative PCR and the 2^-ΔΔCt^ method. Methods. 2001;25(4):402–408. doi: 10.1006/meth.2001.1262. 11846609

[22] Messeguer X, Escudero R, Farré D, et al. PROMO: detection of known transcription regulatory elements using species-tailored searches. Bioinformatics. 2002;18(2):333–334. PMID: 11847087. doi: 10.1093/bioinformatics/18.2.333. 11847087

[23] Farré D, Roset R, Huerta M, et al. Identification of patterns in biological sequences at the ALGGEN server: PROMO and MALGEN. Nucleic Acids Res. 2003;31(13):3651–3653. doi: 10.1093/nar/gkg605. 12824386 PMC169011

[24] Palmiere C, Mangin P. Postmortem biochemical investigations in hypothermia fatalities. Int J Legal Med. 2013;127(2):267–276. PMID: 22773274. doi: 10.1007/s00414-012-0738-y. 22773274

[25] Kaija H, Pakanen L, Porvari K. RNU6B, a frequent reference in miRNA expression studies, differentiates between deaths caused by hypothermia and chronic cardiac ischemia. Int J Legal Med. 2020;134(1):159–162. doi: 10.1007/s00414-019-02041-0. 30904931 PMC6949321

[26] Kaija H, Pakanen L, Porvari K. Low myocardial transcript variant alt-a of cyclin dependent kinase inhibitor p21 expression differentiates hypothermia from cardiac/respiratory causes of death. Medicine. 2020;99(9):e19399. doi: 10.1097/MD.0000000000019399. 32118793 PMC7478380

[27] Chen JH, Michiue T, Ishikawa T, et al. Molecular pathology of natriuretic peptides in the myocardium with special regard to fatal intoxication, hypothermia, and hyperthermia. Int J Legal Med. 2012;126(5):747–756. doi: 10.1007/s00414-012-0732-4. 22752749

[28] Umehara T, Murase T, Abe Y, et al. Identification of potential markers of fatal hypothermia by a body temperature-dependent gene expression assay. Int J Legal Med. 2019;133(2):335–345. doi: 10.1007/s00414-018-1888-3. 29959558

[29] Takamiya M, Saigusa K, Dewa K. DNA microarray analysis of the mouse adrenal gland for the detection of hypothermia biomarkers: potential usefulness for forensic investigation. Ther Hypothermia Temp Manag. 2013;3(2):63–73. doi: 10.1089/ther.2013.0001. 23781398 PMC3684135

[30] Umehara T, Kagawa S, Tomida A, et al. Body temperature-dependent microRNA expression analysis in rats: *rno-miR-374-5p* regulates apoptosis in skeletal muscle cells via *Mex3B* under hypothermia. Sci Rep. 2020;10(1):15432. doi: 10.1038/s41598-020-71931-w. PMC7508983 32963265

[31] Sun Y, Stenson K, Mohan ML, et al. Hypoxia sensing of β-adrenergic receptor is regulated by endosomal PI3Kγ. Circ Res. 2023;132(6):690–703. doi: 10.1161/CIRCRESAHA.122.321735. 36779349 PMC10023460

[32] Cahill T, Chan S, Overton IM, et al. Transcriptome profiling reveals enhanced mitochondrial activity as a cold adaptive strategy to hypothermia in zebrafish muscle. Cells. 2023;12(10):1366. doi: 10.3390/cells12101366. 37408201 PMC10216211

[33] Koeppen M, Lee JW, Seo SW, et al. Hypoxia-inducible factor 2-alpha-dependent induction of amphiregulin dampens myocardial ischemia-reperfusion injury. Nat Commun. 2018;9(1):816. doi: 10.1038/s41467-018-03105-2. 29483579 PMC5827027

[34] Grzebyk E, Pazgan-Simon M, Jagas J, et al. Left ventricular function is related with amphiregulin and fibrosis markers in cirrhotic cardiomyopathy. J Physiol Pharmacol. 2022;73(1):97–107. PMID: 35793762 doi: 10.26402/jpp.2022.1.10. 35793762

[35] Adu-Amankwaah J, Adzika GK, Adekunle AO, et al. ADAM17, a key player of cardiac inflammation and fibrosis in heart failure development during chronic catecholamine stress. Front Cell Dev Biol. 2021;9:732952. doi: 10.3389/fcell.2021.732952. 34966735 PMC8710811

[36] Ji M, Liu Y, Zuo Z, et al. Downregulation of amphiregulin improves cardiac hypertrophy via attenuating oxidative stress and apoptosis. Biol Direct. 2022;17(1):21. doi: 10.1186/s13062-022-00334-w. 35996142 PMC9394079

[37] Dede S, Deger Y, Meral I. Effect of short-term hypothermia on lipid peroxidation and antioxidant enzyme activity in rats. J Vet Med A Physiol Pathol Clin Med. 2002;49(6):286–288. doi: 10.1046/j.1439-0442.2002.00449.x. 12227469

[38] Magni F, Panduri G, Paolocci N. Hypothermia triggers iron-dependent lipoperoxidative damage in the isolated rat heart. Free Radic Biol Med. 1994;16(4):465–476. doi: 10.1016/0891-5849(94)90124-4.ISSN 08915849. 8005532

[39] Anderson EJ, Katunga LA, Willis MS. Mitochondria as a source and target of lipid peroxidation products in healthy and diseased heart. Clin Exp Pharmacol Physiol. 2012;39(2):179–193. doi: 10.1111/j.1440-1681.2011.05641.x. 22066679 PMC3827773

